# An Infant Born with Teeth

**Published:** 1893-01

**Authors:** 


AN INFANT BORN WITH TEETH.—J. Kaufman, M. D, in *Medical Record.*—The following curious case of an infant born at the 7th month of pregnancy with a full set of teeth I think worthy to be recorded. "I delivered Mrs. M. B., a primipira, aged 17, strong and robust, in her seventh month of pregnancy of a male child The labor was rather tedious and the child was born asphyxiated. On introducing my finger into the child's mouth a sensation of teeth was imparted to it from the upper and lower jaw. I did not pay much attention to this at the time, being occupied with the resuscitation of the child, in which I succeeded. The next day finding that the child refused to take the breast, I examined its mouth and to my utter astonishment I found a full set of teeth in the upper and lower maxillæ. The mother, on being told of this, remarked that she had dreamed it the night before".

